# Long-term mesh-related complications from minimally invasive intraperitoneal onlay mesh for small to medium-sized ventral hernias

**DOI:** 10.1007/s00464-024-10716-y

**Published:** 2024-02-29

**Authors:** Sara M. Maskal, Ryan C. Ellis, Ouen Mali, Braden Lau, Nir Messer, Xinyan Zheng, Benjamin T. Miller, Clayton C. Petro, Ajita S. Prabhu, Michael J. Rosen, Lucas R. A. Beffa

**Affiliations:** 1 Center for Abdominal Core Health, Cleveland Clinic, 2049 E 100th St, Desk A-100, Cleveland, OH 44106 USA; 2 Weill Cornell Medicine, New York, USA

**Keywords:** Intraperitoneal mesh, IPOM, Incisional hernia repair, Incisional hernia, Outcomes, Mesh-related complications

## Abstract

**Introduction:**

Intraperitoneal onlay mesh (IPOM) placement for small to medium-sized hernias has garnered negative attention due to perceived long-term risk of mesh-related complications. However, sparse data exists supporting such claims after minimally invasive (MIS) IPOM repairs and most is hindered by the lack of long-term follow-up. We sought to report long-term outcomes and mesh-related complications of MIS IPOM ventral hernia repairs.

**Methods and procedures:**

Adult patients who underwent MIS IPOM ventral hernia repair at our institution were identified in the Abdominal Core Health Quality Collaborative database from October 2013 to October 2020. Outcomes included hernia recurrence and mesh-related complications or reoperations up to 6 years postoperatively.

**Results:**

A total of 325 patients were identified. The majority (97.2%) of cases were elective, non-recurrent (74.5%), and CDC class I (99.4%). Mean hernia width was 4.16 ± 3.86 cm. Median follow-up was 3.6 (IQR 2.8–5) years. Surgeon-entered or patient-reported follow-up was available for 253 (77.8%) patients at 3 years or greater postoperatively. One patient experienced an early small bowel obstruction and was reoperated on within 30 days. Two-hundred forty-five radiographic examinations were available up to 6 years postoperatively. Twenty-seven patients had hernia recurrence on radiographic examination up to 6 years postoperatively. During long-term follow-up, two mesh-related complications required reoperations: mesh removed for chronic pain and mesh removal at the time of colon surgery for perforated cancer. Sixteen additional patients required reoperation within 6 years for the following reasons: hernia recurrence (*n* = 5), unrelated intraabdominal pathology (*n* = 9), obstructed port site hernia (*n* = 1), and adhesive bowel obstruction unrelated to the prosthesis (*n* = 1). The rate of reoperation due to intraperitoneal mesh complications was 0.62% (2/325) with up to 6 year follow-up.

**Conclusion:**

Intraperitoneal mesh for repair of small to medium-sized hernias has an extremely low rate of long-term mesh-related complications. It remains a safe and durable option for hernia surgeons.

Although laparoscopic and robotic ventral hernia repair are commonly performed in the United States, concerns have been raised over the performance of mesh within the peritoneal cavity and the potential for mesh-related catastrophic complications. Current expert guidelines favor retromuscular placement of mesh over intraperitoneal mesh [1], which has led to significant increase in the use of extraperitoneal mesh for several reasons including minimizing mesh-bowel interaction, avoidance of mesh fixation, use of a non-coated mesh, and improved ability to withstand mesh infections [2–4]. For large hernia defects, reconstruction of the abdominal wall with component separations remains and should be the mainstay of treatment. However, the current guidelines fail to distinguish when a component separation is needed and thus these recommendations are being interpreted for all hernias regardless of size. This has led to a decreased in the incidence of laparoscopic intraperitoneal onlay mesh (IPOM) for incisional hernia repairs from 33.8 to 21% with a corresponding increase in retromuscular approaches from 32.1 to 41.4% between 2013 and 2019 [4]. There remains little objective data evaluating the risk of mesh-related complications following minimally invasive (MIS) IPOM repairs particularly for small to medium-sized defects to justify the risk of attempting to get the mesh outside the peritoneal cavity.

Several concerns related to intraperitoneal mesh placement have been cited, from increased wound complications to chronic pain, with weak supporting evidence [5–7]. There are limited prior studies reporting long-term mesh-related complications after IPOM repairs, yet more recent outcome reporting has garnered focus on this topic [6]. While there are advantages to retromuscular sublay mesh placement for larger defects, there are also significant potential complications associated with retromuscular dissections including posterior sheath disruption, linea semilunaris injury, and deep mesh infections [8]. These retromuscular techniques that were originally intended to be used for larger defects in order to reconstruct the abdominal wall are increasingly being utilized in smaller to medium-sized hernias due to concern for intraperitoneal mesh-related complications. We aim to evaluate the long-term outcomes and mesh-related complications of patients undergoing minimally invasive IPOM hernia repairs at our institution.

## Methods

After obtaining approval from the Institutional Review Board under exempt status, the Abdominal Core Health Quality Collaborative (ACHQC) was queried. The ACHQC is a surgeon-entered, prospectively collected database that includes granular data regarding patient characteristics, operative details, clinical outcomes, and patient reported [9]. Our query included adult patients from our institution who underwent a laparoscopic or robotic ventral hernia repair for a primary or incisional hernia with intraperitoneal, permanent synthetic mesh placement from October 2013 to October 2020. We excluded open, conversions to open, hybrid procedures, alternate mesh placements, and patients with a stoma in place. Surgeons must regularly input operative details and 30-day follow-up entries to maintain participant status in the ACHQC, but there is no enforcement of long-term data entry, so this is variably complete. The participating surgeons within our institution have substantial support to ensure long-term follow-up is completed for more patients than average participating surgeons, so we elected to include only our institutional data to maximize the robustness of the long-term follow-up data as this was paramount.

Outcomes of interest included mesh-related complications at any point postoperatively, as well as wound morbidity, reoperations, and hernia recurrence based on radiographic exam and pragmatic definition up to 6 years postoperatively [10]. The pragmatic definition accounts for a patient-reported “bulge,” which may be outweighed by a negative clinical or radiographic examination. Abdominal wall-specific quality of life was measured using the HerQLes survey, which is scored from 0 to 100 with higher scores indicating better quality of life and a minimal clinically important difference of 15.6 [11, 12]. Pain was measured using the NIH PROMIS 3a Pain Intensity Scores, which is scored from 30.7 to 71.8 with lower scores indicating less pain. The lowest possible score on the PROMIS 3a scale is 30.7 indicating “no pain” [13]. The HerQLes survey, PROMIS, and questions regarding a “bulge” or reoperation are routinely included in patient-reported questionnaires which are available for patients to complete electronically or via telephone at preoperative baseline and annually up to 6 years postoperatively. Categorical variables were described using counts and percentages. Continuous variables were described using means and standard deviation. Missing data was not imputed.

## Results

**Fig. 1:**
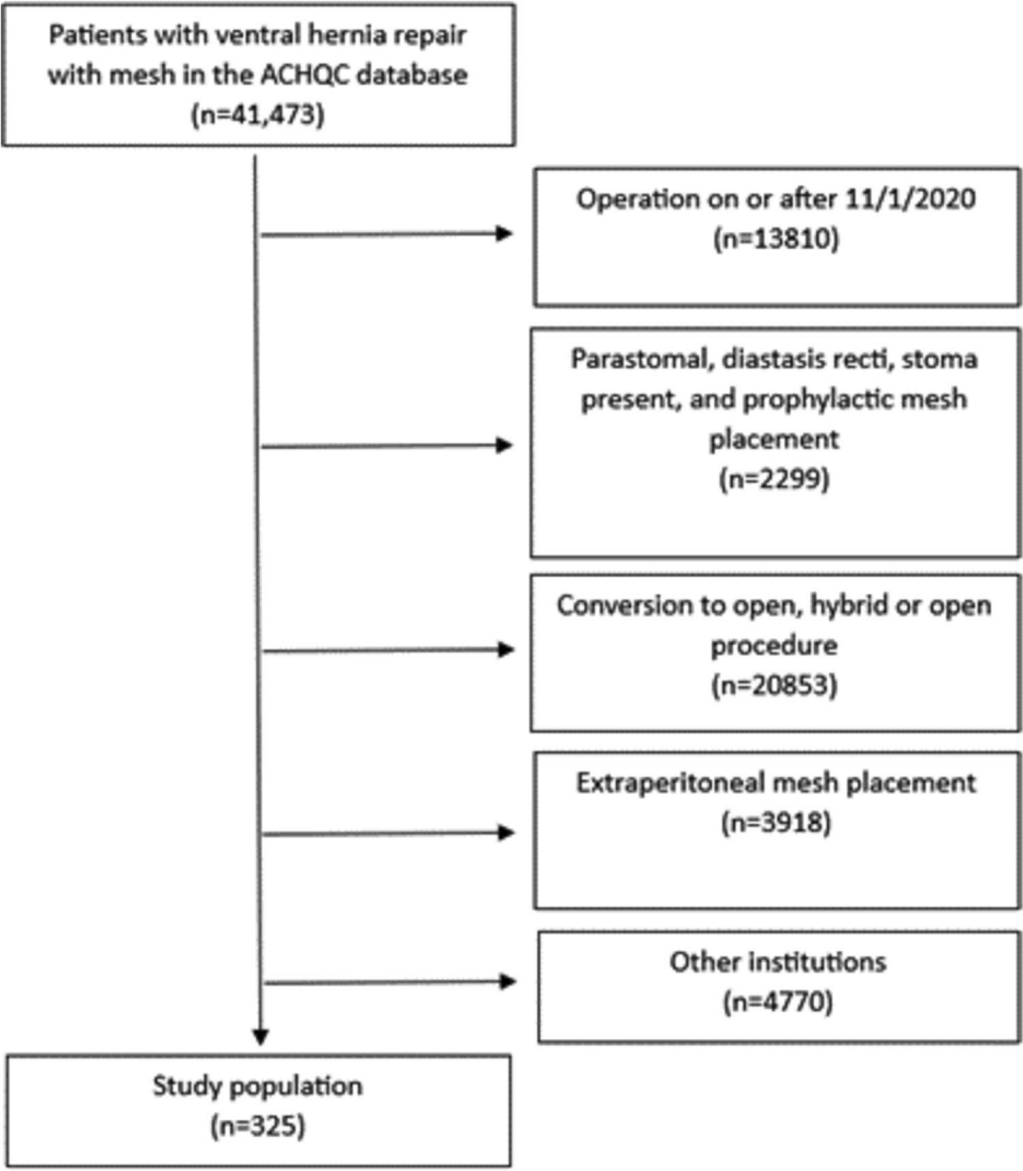
Consort diagram

A total of 325 patients met inclusion criteria (Fig. 1). Mean age was 57.0 ± 13.2 years, mean BMI was 33.1 ± 6.81 kg/m^2^, and 50.2% were female. Patient characteristics are summarized in Table 1. The majority (97.2%) of cases were elective, non-recurrent (74.5%), and CDC class I (99.4%). Average hernia width was 4.16 ± 3.86 cm. Fascia was closed for 203 (62.5%) patients. Operative details are summarized in Table 2.

**Table 1:** Demographics

	Overall (*N* = 325)
Age (years), mean (SD)	57.0 (13.2)
Female, *n*(%)	163 (50.2%)
White race, *n*(%)	267 (83.4%)
BMI (kg/m^2^), mean (SD)	33.1 (6.81)
Recurrent hernia, *n*(%)	83 (25.5%)
Hypertension, *n*(%)	168 (51.7%)
Diabetes mellitus, *n*(%)	63 (19.4%)
Hepatic insufficiency or liver failure, *n*(%)	2 (0.6%)
COPD, *n*(%)	29 (8.9%)
Inflammatory bowel disease, *n*(%)	6 (1.8%)
Anti-platelet medications, *n*(%)	20 (6.2%)
Anti-coagulation medications, *n*(%)	13 (4.0%)
Immunosuppressants, *n*(%)	32 (9.8%)
History of abdominal wall SSI, *n*(%)	13 (4.0%)

*COPD* chronic obstructive pulmonary disease

**Table 2:** Operative details

	Overall (*N* = 325)
Elective case, *n*(%)	316 (97.2%)
ASA class, *n*(%)	
I	3 (0.9%)
II	99 (30.5%)
III	210 (64.6%)
IV	13 (4.0%)
Operative approach, *n*(%)	
Laparoscopic	236 (72.6%)
Robotic	89 (27.4%)
Wound status, *n*(%)	
Clean	323 (99.4%)
Clean-contaminated	1 (0.3%)
Contaminated	1 (0.3%)
Operative time (minutes), *n*(%)	
0–59	99 (30.5%)
60–119	137 (42.2%)
120–179	72 (22.2%)
180–239	9 (2.8%)
240 +	8 (2.5%)
EHS hernia classification, *n*(%)	
M1	20 (6.2%)
M2	164 (50.5%)
M3	206 (63.4%)
M4	52 (16.0%)
M5	7 (2.2%)
Non-midline	14 (4.3%)
Concomitant procedure performed, *n*(%)	21 (6.5%)
Other hernia	8
Foregut/endocrine	3
Hepatobiliary/pancreatic	2
Obstetric/gynecologic	2
Urologic	1
Soft tissue/plastics	6
Intra-operative complications, *n*(%)	8 (2.5%)
Bowel injury	6
Liver injury	1
Unspecified	1
Hernia length (cm), mean (SD)	5.35 (4.07)
Hernia width(cm), mean (SD)	4.16 (3.86)
Mesh length (cm), mean (SD)	15.7 (4.73)
Mesh width(cm), mean (SD)	14.2 (3.18)
Fascial closure, *n*(%)	203 (62.5)

*ASA* American Society of Anesthesiologists, *EHS* European Hernia Society

Median follow-up was 3.6 (IQR 2.8–5) years. One hundred sixty-eight (51.7%) patients had a surgeon-entered follow-up at 3 years postoperatively or greater, which included a clinical exam, radiographic imaging, or both. There was surgeon-entered or patient-reported follow-up available for 253 (77.8%) patients total at 3 years or greater. There was one reoperation within 30 days postoperatively for an early small bowel obstruction, which the surgeon attributed to intraabdominal adhesions unrelated to the mesh. During long-term follow-up, two mesh-related complications required reoperations: mesh removed for chronic pain without alternative explanation (*n* = 1), and mesh removal at the time of colon surgery for perforated cancer (*n* = 1). Sixteen additional patients required reoperation within 6 years postoperatively for the following reasons: hernia recurrence (*n* = 5), unrelated intraabdominal pathology (*n* = 9), obstructed port site hernia (*n* = 1), and adhesive bowel obstruction clearly unrelated to the prosthesis (*n* = 1). Overall, the rate of reoperation due to intraperitoneal mesh complications was 0.62% (2/325) with up to 6 year follow-up (Table 3).

**Table 3:** Long-term outcomes

	*N*	*n*(%)
1 year		
SSI	97	0
SSO	97	3 (3.1%)
SSOPI	97	1 (1.0%)
Reoperation	97	4 (4.1%)
Radiographic recurrence	82	7 (7.2%)
Pragmatic recurrence	155	16 (10.3%)
2 year		
SSI	37	0
SSO	37	0
SSOPI	37	0
Reoperation	37	2 (5.4%)
Radiographic recurrence	36	7 (18.9%)
Pragmatic recurrence	110	25 (22.7%)
3 year		
SSI	57	0
SSO	57	0
SSOPI	57	0
Reoperation	57	5 (8.8%)
Radiographic recurrence	47	6 (10.5%)
Pragmatic recurrence	129	
4 year		
SSI	45	0
SSO	45	0
SSOPI	45	0
Reoperation	45	4 (8.9%)
Radiographic recurrence	38	2 (4.4%)
Pragmatic recurrence	89	11 (12.4%)
5 year		
SSI	32	0
SSO	32	1 (3.1%)
SSOPI	32	0
Reoperation	32	2 (6.3%)
Radiographic recurrence	27	3 (9.4%)
Pragmatic recurrence	55	7 (12.7%)
6 year		
SSI	21	0
SSO	21	0
SSOPI	21	0
Reoperation	21	2 (9.5%)
Radiographic recurrence	15	0
Pragmatic recurrence	34	8 (23.5%)

*SSI* surgical site infection, *SSO* surgical site occurrence, *SSOPI* surgical site occurrence requiring surgical intervention

**Fig. 2:**
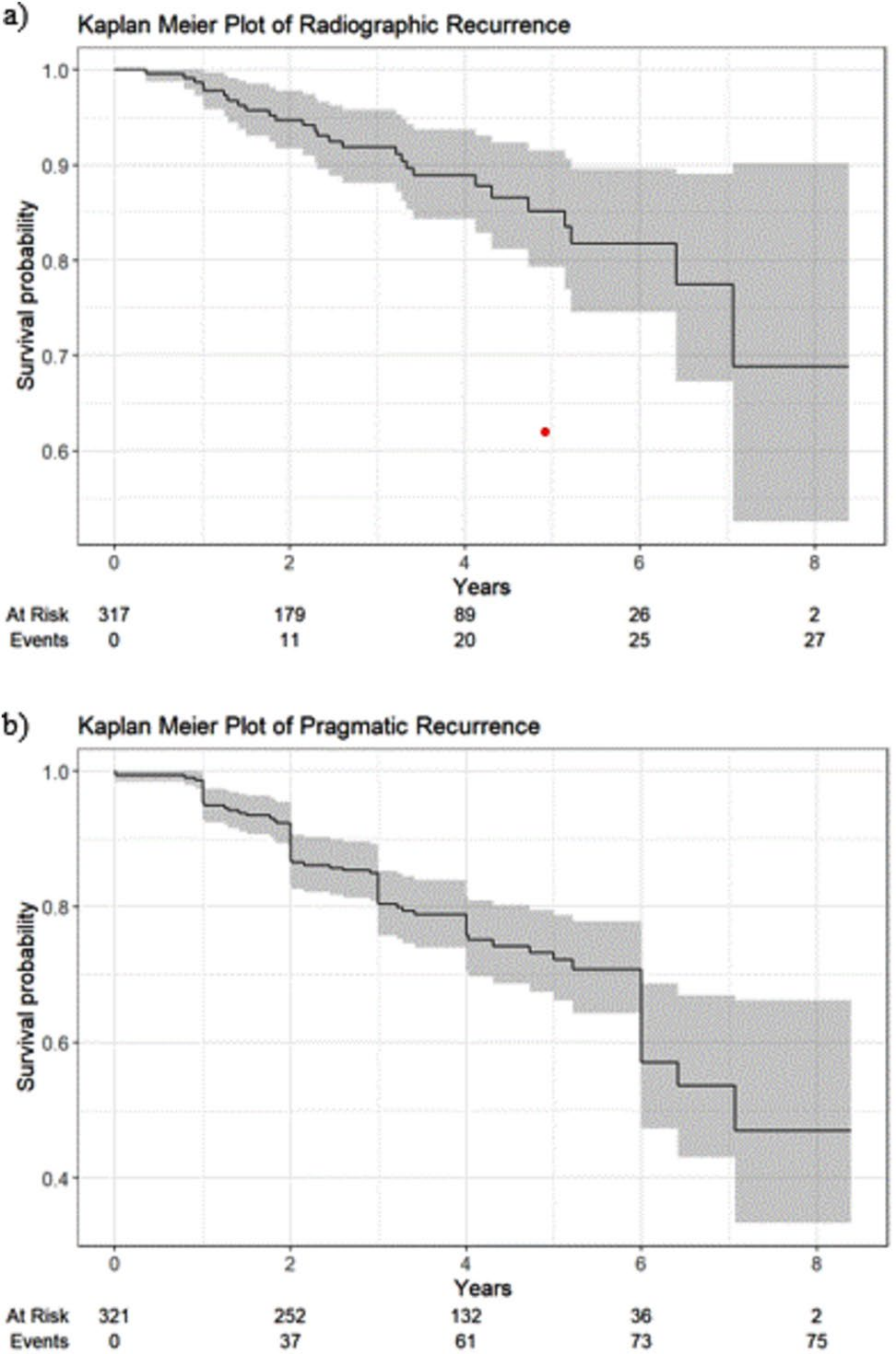
Hernia recurrence by radiographic (**a**) and pragmatic (**b**) definitions

**Fig. 3:**
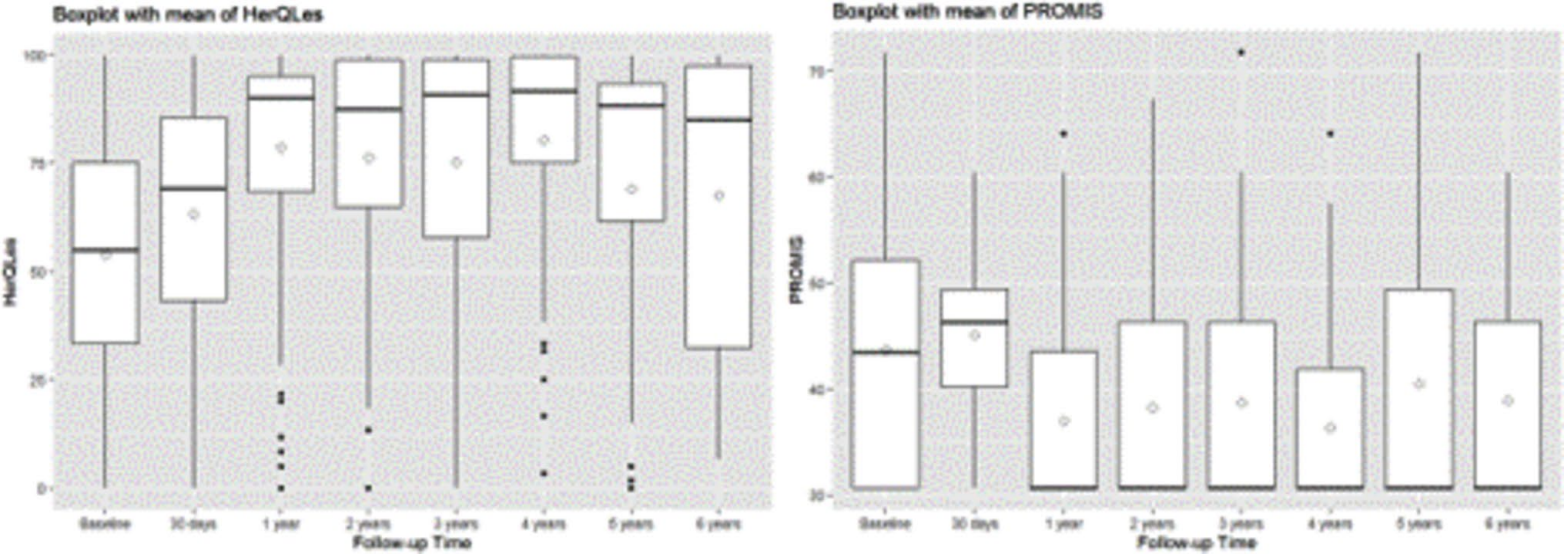
Quality of life (HerQLes) and pain outcomes (PROMIS)

Two hundred forty-five radiographic examinations were available for 129 patients between 1 and 6 years. Twenty-seven patients had a hernia recurrence on radiographic examination between 1 and 6 year follow-up (Fig. 2). Based on the pragmatic definition, 75 patients had a hernia recurrence between 1 and 6 year follow-up (Fig. 2). Median HerQles score improved from 55 (IQR 33.3, 75) at baseline to 90 (IQR 68.3, 95) at 1 year and 85 (IQR 32.5, 97.5) at 6 years postoperatively. Median PROMIS scores improved from 43.5 (IQR 30.7, 52.1) at baseline to 30.7 (IQR 30.7, 43.5) at 1 year and 30.7 (IQR 30.7, 46.3) at 6 years postoperatively (Fig. 3).

## Discussion

In this long-term follow-up study of a single-center review of MIS IPOM ventral hernia repairs with a median follow-up of 3.6 years, we found that mesh-related complications and reoperations were extremely rare, less than 1%. We also found no long-term incidence of chronic wounds or bowel erosions in this cohort. Radiographic hernia recurrence was consistent with other studies at ~ 8% [14–16]. Quality of life scores improved significantly and remained improved over the follow up period. This data suggests that IPOM ventral hernia repairs for small to medium-sized hernias should still be considered a safe and durable approach.

The ideal position for mesh placement in ventral hernia repairs remains debated. There has been a movement away from intraperitoneal mesh placement in the surgical community, which is reflected in expert consensus-based guidelines endorsing retromuscular mesh placement as the preferred approach despite acknowledging weak evidence [1]. The decision of which layer of abdominal wall to place mesh is complex decision and requires a tailored approach based on multiple factors including hernia morphology, patient physiology, and risk profile. Each mesh location carries a unique risk profile that warrants consideration, yet IPOM repairs have been shunned in recent years for various perceived downsides, each of which we will address, including: mesh-related complications, perception of worse intra-abdominal adhesions, hernia recurrence, and pain.

One concern regarding IPOM repair is mesh-related complications including long-term wound morbidity (sinus tracts, chronic mesh infections) or bowel erosions resulting in enteroprosthetic fistula formation. This stems from apprehension of placing prosthetic material in direct contact with bowel [5]. This has been challenged by large retrospective cohort studies showing similar SSI and SSO rates between intraperitoneal and extraperitoneal mesh placements without fistula formation [17–21]. Additionally, multiple randomized trials, meta-analyses, and systematic reviews comparing open and laparoscopic IPOM repairs for ventral hernias, demonstrate that laparoscopy has equivalent or fewer wound complications, although follow up has been limited [22–24]. In a recent randomized trial of robotic eTEP versus IPOM repairs of similarly sized hernias, wound complication rates were similar between approaches at 1 year follow-up [7, 25]. The long-term data presented in this work supports that minimally invasive IPOM have extremely small risk of mesh-related chronic wound complications or fistula formation up to 6 years after IPOM considering that none occurred in this cohort.

Intraperitoneal mesh is commonly thought to stimulate significant intra-abdominal adhesions, which raises concerns for future operations. Gray et al. reported a four-fold increased risk of reoperation with intraperitoneal mesh placement [26]. It should be emphasized that this report included mostly uncoated mesh in the intraperitoneal position, which is an antiquated practice. As a solution to this, barrier coated meshes were developed and employed for use in the intraperitoneal position demonstrating safety and low incidence of mesh-related complications [27]. Chelala et al. reported compelling long-term follow-up of 1326 patients who underwent IPOM repairs with a mean follow-up of 78 months, of which 126 underwent reoperations for various reasons. Adhesions were graded during reoperation and ~ 90% were classified as Mueller 0-I (adhesion free or having loose omental adhesions only [16]. Hayden et al. similarly reported no difference in adhesion formation or complications during reoperation based on mesh placement in a retrospective cohort of 433 patients [14]. Our data is congruent with these trials demonstrating mesh-related complications at reoperation is rare.

IPOM repairs have drawn criticism due to studies reporting higher rates of hernia recurrence. In a series of 447 patients, Holihan et al. demonstrated a lower rate of hernia recurrence for sublay repairs compared to intraperitoneal (hazard ratio 0.4, 95% CI 0.2–0.8), which concurred with a preceding systematic review and network analysis demonstrating the lowest risk for recurrence with sublay mesh placement [17, 28]. Placement of mesh in a well vascularized retromuscular space should theoretically lead to improved integration of the mesh into the abdominal wall compared to intraperitoneal mesh placement, however, a recent randomized trial showed no difference in hernia recurrence between robotic extraperitoneal and intraperitoneal mesh placement [25]. This difference in recurrence depending on mesh placement may also be explained by selecting the correct size defects for IPOM repairs. In hernias 7 cm or wider, we prefer to not use an IPOM repair, but rather a retromuscular sublay in either a MIS or open approach.

The recurrence rate presented in our dataset was comparable to other trials utilizing IPOM repairs [15, 16, 29–32]. Kokotovic et al. reported similar reoperation rates for hernia recurrence after both open and laparoscopic repairs, however the data source used was a national patient registry which does not include the same granular operative details or surgeon-entered follow-up as the ACHQC [27]. It should be noted that the pragmatic recurrence rate, which incorporates patient-related bulge in the absence of clinical or radiographic follow-up, was significantly higher than the radiographic recurrence rate alone [10, 33]. The prevalence of radiographic follow-up diminished more quickly than the patient-reported outcomes, meaning that the pragmatic definition of recurrence in later years was more heavily influenced by patient perceived bulge than clinical or radiographic evidence of a hernia recurrence. This maximizes sensitivity and data capture, but may overestimate the true incidence of hernia recurrence.

Lastly, there is concern about increased pain following a laparoscopic or robotic IPOM when compared to a retromuscular hernia repair. Higher pain rates in IPOM are often attributed to the need for mesh fixation with tacking devices or due to trans-fascial sutures. Similarly, the lack of need for mesh fixation in retromuscular repairs has been touted as a benefit. There are previous trials that report enhanced-view totally extraperitoneal (eTEP) as a less painful alternative to IPOM [34, 35]. In contrast, the REVEAL randomized controlled trial of 100 patients demonstrated no difference in NRS-11 scores in the immediate postoperative period, but less pain for eTEP compared to IPOM on 30 day PROMIS 3a scale [median, (IQR): 46.3 (43.5–52.1) vs. 43.5 (40.2–49.4), *p* = 0.049] [7]. In 1 year follow-up of the same trial, the difference in pain scores disappeared. Long-term pain scores were minimal in this cohort, which is congruent with other studies reporting low rates of chronic pain with IPOM [36]. Overall, the concern that IPOM is a painful approach should be evaluated in more prospective trials, but appears to be overstated.

The ideal location for mesh is not a singular “one size fits all” approach and must be tailored to the clinical scenario. IPOM and retromuscular repairs both have benefits and consequences which need to be weighed against the risks of the repair itself for the patient. The drive to keep mesh out of the peritoneal cavity may have unintended consequences including linea alba disruption, linea semilunaris injury, and posterior sheath disruption [8]. Considering that transversus abdominis release is also often the last resort for complex hernias, it is also advisable to avoid utilizing the retromuscular plane for routine small to medium-sized hernia. IPOM has been eschewed for various reasons yet remains one of the most well-studied and well-proven repairs for small to medium-sized ventral hernias. Therefore, it is logical to conclude that an MIS IPOM remains a valuable tool in the armamentarium of the modern-day hernia surgeon.

There are multiple limitations to address in this study. As a retrospective database query, follow up is always challenging and patients have been selected to have MIS IPOM repairs thus introducing selection biases. We did not restrict analysis to incisional hernias, which may impact hernia recurrence rates. The lack of radiographic and clinical follow-up was supplemented by patient-reported outcomes, which increases the capture of outcomes, but may also hyperinflate the incidence of hernia recurrence given the high sensitivity of the Hernia Recurrence Inventory [33]. As a descriptive series, there was no comparator group, which limits our ability to suggest relative safety or efficacy of intraperitoneal mesh placement as opposed to alternative placements. Finally, the lack of generalizability of this data should be considered as these surgeries were performed by surgeons in a high-volume center with advanced training in both abdominal wall reconstruction and minimally invasive surgery techniques.

## Conclusion

Minimally invasive IPOM remains a safe and durable option for small to medium-sized hernias with a low incidence of long-term mesh-related complications in our experience. The avoidance of IPOM repairs due to concern for catastrophic complications should be discouraged.
